# Privacy assurances and professional-boundary warnings in generative AI mental health chatbots: a randomized vignette experiment on calibrated trust, overreliance risk, and professional help-seeking intentions

**DOI:** 10.3389/fpsyg.2026.1934264

**Published:** 2026-09-01

**Authors:** Zeyu Zhang, Xiaomei Lu, Yinlan Zhang, Hao Zhang, Min Zhang

**Affiliations:** 1 Judicial Police College, Xinjiang University of Political Science and Law, Tumxuk, China; 2 Department of Nursing, Sihong Hospital, Suqian, China; 3 Department of Medical Technology, Nanjing Second Hospital, Nanjing, China; 4 Department of General Surgery, Suqian Hospital of Traditional Chinese Medicine, Suqian, China; 5 Teaching and Research Group, Suqian Chongwen Middle School, Suqian, China

**Keywords:** calibrated trust, generative AI chatbot, mental health support, overreliance risk, privacy assurance, professional-boundary warning

## Abstract

**Introduction:**

Generative artificial intelligence (AI) chatbots are increasingly used by students to look up mental health information, seek reassurance, explore coping strategies, and identify possible sources of support. In such sensitive contexts, however, their use raises concerns about privacy, professional boundaries, and the risk of relying on AI beyond its proper role. This study examined whether two brief interface messages-privacy assurance and professional-boundary warning-affect students' safety-related evaluations of generative AI mental health chatbots.

**Methods:**

We conducted a 2 × 2 randomized vignette experiment with 768 college students. Participants were assigned to one of four chatbot scenarios that either included or omitted privacy assurance and professional-boundary warning. Measures covered perceived privacy protection, boundary awareness, calibrated trust, safe-use intention, overreliance risk, and professional help-seeking intention.

**Results:**

Privacy assurance increased perceived privacy protection, *F*(1, 764) = 159.30, *p* < 0.001, ηp^2^ = 0.172, *d* = 0.91. Professional-boundary warning increased boundary awareness, *F*(1, 764) = 176.40, *p* < 0.001, ηp^2^ = 0.188, *d* = 0.96. The structural model showed good fit, χ^2^(302) = 742.65, CFI =0.955, TLI = 0.948, RMSEA = 0.044, and SRMR = 0.046. Perceived privacy protection and boundary awareness were associated with calibrated trust. Calibrated trust and perceived privacy protection were associated with safe-use intention, whereas boundary awareness was linked to lower overreliance risk and stronger professional help-seeking intention. Calibrated trust was highest when both messages were present.

**Discussion:**

These findings suggest that visible privacy and boundary messages can influence how students judge AI chatbots in mental health-related situations. Such messages should not be understood as prompts for greater use. Rather, they may help users treat chatbots as limited tools for information and support navigation and recognize when professional help is needed.

## 1 Introduction

### 1.1 Generative AI chatbots and mental health support

Generative artificial intelligence (AI) chatbots have rapidly become part of students' everyday information-seeking and communication practices. Their conversational interface, immediate response capacity, and ability to generate personalized text have made them attractive as low-threshold tools for academic, social, and health-related questions. In higher education, AI chatbots have been studied primarily in relation to adoption, perceived usefulness, satisfaction, behavioral intention, creativity, and learning support (Hwang and Wu, 2025; Tian et al., 2024). Yet their growing use also extends beyond academic tasks, including situations in which students ask for emotional reassurance, coping suggestions, or guidance about whether and how to seek mental health support.

In mental health-related contexts, the potential appeal of generative AI chatbots is easy to understand. Students may hesitate to disclose distress to peers, teachers, or professionals because of stigma, uncertainty, time constraints, or lack of access to services. A chatbot can appear available, non-judgmental, and easy to approach. Digital mental health research has long noted that technology-mediated services may reduce access barriers and provide timely forms of support (Lal and Adair, 2014; Lattie et al., 2019). Earlier reviews of mental health chatbots have summarized their common features, perceived benefits, user concerns, and safety-related limitations, while also emphasizing the need for clearer evidence on appropriate use (Abd-Alrazaq et al., 2019, 2021; Vaidyam et al., 2019). Early chatbot studies also suggested that conversational agents can provide structured psychoeducation and supportive interaction under specific conditions (Fitzpatrick et al., 2017; Fulmer et al., 2018).

However, the emergence of generative AI systems changes the risk profile of chatbot-based support. Unlike earlier rule-based or scripted conversational agents, large language models can generate fluent and context-sensitive responses across a very wide range of topics. Fluency may increase perceived competence, but it may also obscure uncertainty, hallucinated information, or the lack of professional accountability. In mental health-related settings, this creates a delicate tension: AI chatbots may serve as sources of general information and support navigation, but they should not be treated as diagnostic, psychotherapeutic, crisis-intervention, or medical decision-making tools (Olisaeloka et al., 2026; Palmer and Schwan, 2025; Pichowicz et al., 2025; Szoke et al., 2026; World Health Organization, 2025).

Recent discussions of AI and student wellbeing emphasize this dual character. AI can improve access, personalization, and communication efficiency, but it may also produce technostress, digital fatigue, privacy concerns, loneliness, and overreliance when users treat automated systems as primary sources of support (Klimova and Pikhart, 2025). These concerns are especially salient for students, who frequently combine academic pressure, social adjustment, and developmental transitions. Large-scale college student mental health research has also shown that mental health problems are common in university populations, making appropriate help-seeking pathways particularly important (Auerbach et al., 2018). Therefore, research should move beyond whether students are willing to use AI chatbots and examine which design cues can support responsible, privacy-sensitive, and professionally bounded use.

### 1.2 Safety-design cues: privacy assurance and professional-boundary warning

The present study focuses on two safety-design cues that can be embedded in AI chatbot interfaces or introductory messages: privacy assurance and professional-boundary warning. Privacy assurance refers to a clear statement that informs users how mental health-related information is handled, protected, and limited in use. In the context of psychological disclosure, privacy is not a secondary usability feature; it is a condition for whether users feel safe enough to interact with a digital support tool. Classic information privacy research shows that perceived data control, transparency, and protection shape user trust and technology acceptance (Belanger and Crossler, 2011; Malhotra et al., 2004; Smith et al., 1996). Recent work on general-purpose LLM chatbots for mental health also suggests that users may misunderstand the privacy and regulatory protections attached to emotionally sensitive disclosures (Kwesi et al., 2025). In health-related AI systems, these issues become more sensitive because users may disclose distress, family problems, sleep problems, interpersonal conflict, or other personal experiences. This emphasis on visible safeguards is consistent with human-centered and trustworthy AI principles, which stress privacy protection, transparency, safety, accountability, and responsible governance (High-Level Expert Group on Artificial Intelligence, 2019; National Governance Committee for the New Generation Artificial Intelligence, 2019; NIST, 2023; Shneiderman, 2020; World Health Organization, 2025).

Professional-boundary warning refers to a clear statement that the chatbot is not a substitute for qualified mental health care, clinical diagnosis, psychotherapy, crisis intervention, or emergency services. Such a cue does not simply reduce expectations; it clarifies the appropriate role of the system. In responsible digital mental health support, a chatbot may provide general information, coping suggestions, reflective prompts, or referral guidance, but professional help is needed when distress is severe, persistent, risky, or functionally impairing. A boundary warning may therefore increase users' awareness of the limits of AI-generated support and help prevent inappropriate reliance.

Together, the two cues target different risks: unsafe disclosure and inappropriate reliance. Privacy assurance addresses whether it is reasonable to share sensitive information, whereas a boundary message clarifies how far chatbot support should go. Their combination may help students approach AI chatbots as educational and support-navigation tools rather than substitutes for qualified care (Olisaeloka et al., 2026; Palmer and Schwan, 2025; World Health Organization, 2025).

### 1.3 Calibrated trust and responsible AI use

Trust is a central construct in human-AI interaction, but in safety-sensitive contexts the goal is not maximum trust. Trust should be calibrated to the actual capabilities and limits of the system. Research on interpersonal trust, online trust, trust in automation, and human-AI trust suggests that trust arises from characteristics of the user, the system, and the interaction context (Glikson and Woolley, 2020; Hoff and Bashir, 2015; Kohn et al., 2021; Lee and See, 2004; Li et al., 2024; Mayer et al., 1995; McKnight et al., 2002). In AI settings, inappropriate trust can produce two opposite problems: disuse, when users avoid potentially useful tools, and misuse, when users rely on systems beyond their appropriate scope (Parasuraman and Manzey, 2010; Parasuraman and Riley, 1997; Parasuraman et al., 2000).

For AI mental health chatbots, calibrated trust can be defined as a balanced evaluation in which users perceive the chatbot as potentially useful for limited, low-threshold support while recognizing its inability to provide professional clinical judgment, diagnosis, psychotherapy, or crisis intervention. Calibrated trust is therefore different from general trust or simple willingness to use. It includes both a positive appraisal of usefulness and a realistic understanding of boundaries. This distinction is important because an intervention that merely increases trust may unintentionally increase overreliance. By contrast, safety-design cues may promote trust that is cautious, informed, and bounded.

Privacy assurance may increase calibrated trust by reducing uncertainty about the handling of sensitive information. Professional-boundary warning may also increase calibrated trust because it signals transparency and honesty about system limitations. Although a warning might appear to lower trust at first glance, a professionally framed boundary statement may actually make the system seem more responsible and trustworthy. Therefore, the combined presence of privacy assurance and boundary warning may produce the strongest form of calibrated trust.

### 1.4 Overreliance risk and professional help-seeking

Overreliance risk is a critical outcome in AI mental health support. It refers to the possibility that users may rely on chatbot responses when professional help would be more appropriate, follow AI-generated suggestions without critical evaluation, or delay seeking qualified care because the chatbot seems responsive or reassuring. This risk is related to automation bias and inappropriate reliance, but it is especially consequential in mental health because delayed professional help may worsen distress or leave risk unrecognized.

Professional help-seeking intention is the complementary responsible-use outcome. Rather than asking only whether users intend to use an AI chatbot, it is more informative to ask whether they would seek qualified help when AI support is insufficient or when distress becomes severe. Help-seeking research has shown that young people often face barriers such as stigma, low perceived need, uncertainty, and preference for self-reliance (Gulliver et al., 2010; Pretorius et al., 2019; Rickwood et al., 2005). AI tools could either reduce these barriers by offering information and referral guidance, or increase avoidance if users treat chatbots as a replacement for professional services.

The present study therefore conceptualizes responsible AI mental health support as a pattern of outcomes: higher safe-use intention, lower overreliance risk, and stronger professional help-seeking intention. This outcome structure shifts the focus from technology acceptance to health psychology and safety design. It asks not only whether students would use such tools, but also whether they would use them with sufficient attention to privacy, limits, and appropriate help-seeking.

### 1.5 Present study and hypotheses

To examine these issues, we conducted a 2 × 2 randomized vignette experiment among college students. The first factor was privacy assurance, which was either absent or present in the chatbot scenario. The second factor was professional-boundary warning, which was either absent or present. Participants read one scenario and then reported perceived privacy protection, boundary awareness, calibrated trust, safe-use intention, overreliance risk, and professional help-seeking intention. Unlike acceptance-oriented frameworks that focus primarily on perceived usefulness, continuance, or behavioral intention (Bhattacherjee, 2001; Davis, 1989; Venkatesh et al., 2003), this experimental approach allows the study to isolate the effects of specific safety-design cues and examine responsible-use outcomes.

Although the hypotheses focused on direct paths among the experimental cues, psychological mechanisms, and outcomes, the model also allowed theory-based indirect pathways in which perceived privacy protection, boundary awareness, and calibrated trust served as linking mechanisms between interface messages and responsible-use outcomes. These indirect pathways were treated as planned secondary mechanisms rather than as separate causal claims.

**Figure 1:**
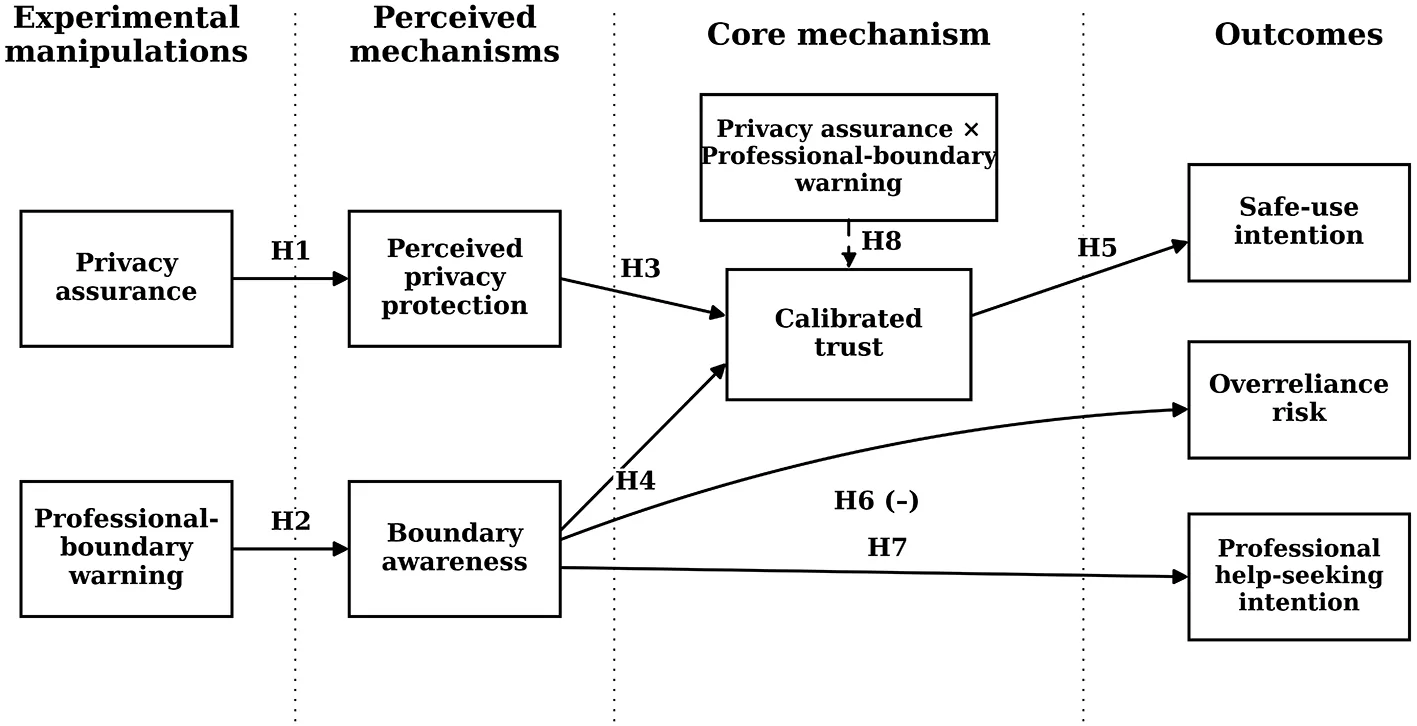
Hypothesized model of responsible use of generative AI mental health chatbots. Solid arrows indicate hypothesized direct effects. The dashed arrow indicates the interaction effect of privacy assurance and professional-boundary warning on calibrated trust. The path from boundary awareness to overreliance risk is expected to be negative.

We proposed the following hypotheses. H1: Privacy assurance increases perceived privacy protection. H2: Professional-boundary warning increases boundary awareness. H3: Perceived privacy protection positively predicts calibrated trust. H4: Boundary awareness positively predicts calibrated trust. H5: Calibrated trust positively predicts safe-use intention. H6: Boundary awareness negatively predicts overreliance risk. H7: Boundary awareness positively predicts professional help-seeking intention. H8: The combined presence of privacy assurance and professional-boundary warning predicts higher calibrated trust than either cue alone. The hypothesized research framework is presented in Figure 1.

## 2 Materials and methods

### 2.1 Study design

This study was designed as a 2 × 2 randomized vignette experiment. The two manipulated factors were privacy assurance (absent vs. present) and professional-boundary warning (absent vs. present). Participants were randomly assigned to one of four conditions: (1) no privacy assurance and no boundary warning, (2) privacy assurance only, (3) boundary warning only, and (4) both privacy assurance and boundary warning. Each participant read one vignette describing a generative AI chatbot that could provide general mental health information, coping suggestions, emotional reassurance, and help-seeking guidance.

The study was not designed to test the clinical effectiveness of an AI chatbot, and participants did not receive diagnosis, psychotherapy, or crisis support from the system. Instead, the vignette method was used to evaluate how safety-design cues shape perceptions and intentions in a standardized scenario. This design is appropriate for early-stage psychological evaluation of interface messages and risk communication because it allows researchers to manipulate specific design features while holding the broader use context constant (Aguinis and Bradley, 2014; Atzmueller and Steiner, 2010).

### 2.2 Participants and procedure

Participants were college students recruited through university-based online survey distribution channels between March 2026 and April 2026. Eligibility criteria were being at least 18 years old, being currently enrolled in a college or university, and providing electronic informed consent. A total of 832 responses were initially collected. Based on pre-specified eligibility and data-quality criteria, 64 responses were excluded: 7 did not provide informed consent, 9 were from participants younger than 18 years, 8 were from participants who were not currently enrolled in college, 19 failed an instructional attention-check item, 13 had unrealistically short completion time, and 8 showed straight-lining or patterned responding. The final analytic sample consisted of 768 valid responses.

Random assignment was implemented automatically by the online survey platform after eligibility screening and electronic consent. The platform used a built-in randomizer with equal allocation probability across the four vignette conditions, and participants could not preview or choose the assigned condition. Each participant saw only one vignette condition. The final cell sizes were balanced, with 192 participants in each condition. After reading the assigned scenario, participants completed manipulation-check items, measures of the focal constructs, and covariates. The survey ended with a debriefing message emphasizing that AI chatbots cannot replace professional mental health services and that participants experiencing severe distress should contact campus counseling services, qualified mental health professionals, medical institutions, or emergency services.

### 2.3 Vignette conditions

All four vignettes began with the same base description: participants were asked to imagine that their university had introduced a generative AI chatbot that could answer general questions about stress, sleep, study pressure, emotional regulation, and help-seeking resources. The chatbot was described as able to provide general information, coping suggestions, emotional reassurance, and referral guidance. It was not described as a clinical service.

In the privacy-assurance condition, the scenario added a statement explaining that the chatbot would minimize the collection of personal information, protect mental health-related data, avoid using identifiable information for unrelated purposes, and clearly inform users how their information would be handled. In the professional-boundary warning condition, the scenario added a statement explaining that the chatbot was not a substitute for diagnosis, psychotherapy, crisis intervention, or medical treatment, and that users should contact qualified professionals or emergency services when distress is severe or urgent. The full vignette texts are provided in Supplementary Table S2.

### 2.4 Measures

Unless otherwise noted, all focal constructs were measured on 5-point Likert-type response scales ranging from 1 (strongly disagree) to 5 (strongly agree). A 5-point format was selected to reduce response burden in an online vignette experiment while providing sufficient variation for construct-level analysis. Items were developed or adapted based on prior work on human-AI trust, privacy concerns, help-seeking, and digital mental health. The item development process followed best-practice recommendations for construct definition, item wording, expert review, target-population feedback, and measurement transparency (Clark and Watson, 2019; Jebb et al., 2021; Stefana et al., 2024). Full focal items, manipulation-check items, and covariate measurement details are shown in Supplementary Table S1.

The focal measures were developed through a multi-step process. First, construct definitions were specified based on prior work on human-AI trust, information privacy, digital mental health, and help-seeking. Second, an initial item pool was generated to reflect the specific context of generative AI mental health chatbots. Third, the items were reviewed by researchers with backgrounds in psychology, digital health, and survey methodology to evaluate content relevance, clarity, and redundancy. Fourth, a small group of students reviewed the wording for comprehensibility before the formal survey. The final items were retained when they clearly represented the intended construct and avoided clinical claims about diagnosis, psychotherapy, or treatment. Full items and scoring details are provided in Supplementary Table S1.

Perceived privacy protection was measured with four items assessing whether the chatbot appeared to protect users' mental health-related information, explain data handling, and provide sufficient privacy safeguards. Boundary awareness was measured with five items assessing whether participants understood that the chatbot had professional limits, was not a substitute for qualified care, and should not be used for crisis intervention. Calibrated trust was measured with five items assessing balanced confidence in the chatbot as a limited support tool and awareness of its limitations.

Safe-use intention was measured with four items assessing willingness to use the chatbot as a supplementary, low-threshold source of general support in appropriate situations. Overreliance risk was measured with four items assessing the possibility of following chatbot advice without professional verification, relying on the chatbot when professional help would be more appropriate, delaying help-seeking because the chatbot seemed helpful, or treating chatbot responses as professional advice. Professional help-seeking intention was measured with four items assessing willingness to contact qualified professionals, campus counseling services, or emergency services when AI support is insufficient or distress becomes severe.

Manipulation checks were included to assess whether participants perceived the intended safety cues. The privacy manipulation check consisted of three items assessing whether the vignette clearly explained information protection and data handling. The boundary manipulation check consisted of three items assessing whether the vignette clearly stated the chatbot's limits and the need for professional help in high-risk situations. Consistent with methodological caution regarding manipulation checks, these items were treated as checks of cue perception rather than as mediators in the primary causal model (Hauser et al., 2018).

Covariates included gender, age, grade, prior generative AI use, prior mental health education, mental health literacy, and psychological distress. Prior GenAI use was assessed with a single self-report item on frequency of use, and prior mental health education was coded as whether participants had previously received formal mental health education or training. Mental health literacy was measured with a brief self-report composite assessing knowledge of common mental health problems, help-seeking resources, reliable information evaluation, and when professional support is needed. Psychological distress was measured with a brief self-report composite assessing recent nervousness, low mood, sleep-related distress, and concentration difficulty. Higher scores indicated higher mental health literacy and greater psychological distress, respectively. These variables were included only as covariates rather than focal theoretical predictors because the present study focused on safety-design cues rather than general willingness mechanisms.

### 2.5 Data quality control

Data quality was addressed through eligibility screening, informed consent, attention checking, completion-time screening, and response-pattern inspection. One instructional attention-check item asked participants to select a specified response option; such checks are commonly used to detect satisficing and inattentive responding in online survey research (Oppenheimer et al., 2009). Because self-report data may still be affected by common method and response-quality concerns, the questionnaire also incorporated procedural safeguards such as anonymity, clear instructions, separation of vignette exposure from outcome items, and response-pattern inspection (Podsakoff et al., 2003). Responses were excluded as unrealistically short if the completion time was below 180 s, which was less than one-third of the median completion time of the valid survey attempts. This criterion was used to identify responses unlikely to reflect careful reading of the vignette and questionnaire items. Straight-lining and patterned responses were also excluded when response patterns suggested non-differentiated answering across conceptually distinct items.

The exclusion criteria were specified before the primary analysis. Analyses were conducted on complete valid cases. Robustness checks re-estimated the main models without covariates, using robust maximum likelihood, using WLSMV for ordinal indicators, excluding frequent GenAI users, and restricting the sample to participants who passed the manipulation checks.

### 2.6 Statistical analysis

An a priori power analysis was conducted using G^*^Power 3.1.9.7 for a 2 × 2 factorial ANOVA. Assuming a small-to-medium effect size, α = 0.05, and power = 0.90, the minimum required sample was approximately 700 participants. To ensure balanced cells after exclusions, the target initial sample was 820–850 participants. The final valid sample of 768 participants exceeded this requirement and provided 192 participants per experimental cell.

First, descriptive statistics and randomization checks were conducted. Second, manipulation checks were analyzed using factorial ANOVA, reporting *F* statistics, degrees of freedom, *p* values, partial eta squared (ηp^2^), and Cohen's d for focal present-vs.-absent comparisons. Third, confirmatory factor analysis (CFA) was used to examine the measurement model. Internal consistency was evaluated using Cronbach's alpha, McDonald's omega, composite reliability (CR), average variance extracted (AVE), and item loadings. Convergent validity was evaluated using CR and AVE (Fornell and Larcker, 1981). Discriminant validity was evaluated primarily using the heterotrait-monotrait ratio (HTMT), which was treated as the main contemporary criterion for detecting insufficient separation between constructs (Henseler et al., 2015). Model fit was evaluated using χ^2^, CFI, TLI, RMSEA, and SRMR, with values of CFI/TLI around 0.95, RMSEA below 0.06, and SRMR below 0.08 interpreted as acceptable to good fit (Hu and Bentler, 1999; Kline, 2023).

Fourth, factorial ANOVA was used to estimate the main and interaction effects of privacy assurance and professional-boundary warning on the focal outcomes. Covariate-adjusted ANCOVA models were conducted as robustness checks. Fifth, structural equation modeling (SEM) tested the hypothesized path model, including standardized path coefficients, standard errors, 95% confidence intervals, *p* values, and *R*^2^ values. CFA and SEM were estimated in Mplus 8.3 using maximum likelihood with robust standard errors, and WLSMV estimation was used as an ordinal-indicator robustness check. Indirect effects were examined to evaluate whether perceived privacy protection, boundary awareness, and calibrated trust served as psychological linking mechanisms between interface messages and responsible-use outcomes. These indirect effects were estimated using bootstrapping with 10,000 resamples, following common recommendations for mediation and conditional process analysis (Hayes, 2022). Descriptive statistics, randomization checks, manipulation checks, and factorial ANOVA were conducted using IBM SPSS Statistics 29.0.

All numerical results reported in the Results section were derived from the final cleaned dataset of 768 valid participants. No simulated or imputed values were used for hypothesis testing.

### 2.7 Ethical considerations

The study protocol was reviewed and approved by the Research Office of Xinjiang University of Political Science and Law, Tumxuk, China. All participants provided electronic informed consent before participation and were informed that they could discontinue the survey at any time without penalty. Because the study involved mental health-related scenarios, the materials clearly stated that the chatbot was not a clinical service and was not intended to provide diagnosis, psychotherapy, crisis intervention, or medical treatment. The survey did not ask participants to describe personal traumatic experiences or disclose current crisis details. At the end of the survey, participants received a debriefing and safety message clarifying that the chatbot scenario was fictional and encouraging them to contact campus counseling services, qualified mental health professionals, medical institutions, or emergency services if they experienced severe distress, thoughts of self-harm, or urgent psychological risk.

## 3 Results

### 3.1 Participant characteristics and randomization check

The final analytic sample included 768 college students, with 192 participants in each of the four experimental conditions. The four groups were comparable in gender, age, grade, prior generative AI use, prior mental health education, mental health literacy, and psychological distress. This pattern indicated that randomization produced balanced groups for the main analyses. Table 1 presents participant characteristics by experimental condition.

**Table 1:** Participant characteristics by experimental condition.

Characteristic	G1 no cues *n* = 192	G2 privacy only *n* = 192	G3 boundary only *n* = 192	G4 both cues *n* = 192	*p*
Age, M (SD)	20.84 (1.42)	20.91 (1.48)	20.76 (1.39)	20.88 (1.45)	0.713
Female, *n* (%)	107 (55.7)	104 (54.2)	106 (55.2)	105 (54.7)	0.991
First/second year, *n* (%)	112 (58.3)	109 (56.8)	114 (59.4)	111 (57.8)	0.963
Prior GenAI use, M (SD)	3.16 (1.08)	3.22 (1.10)	3.18 (1.05)	3.20 (1.09)	0.938
Prior mental health education, *n* (%)	82 (42.7)	85 (44.3)	80 (41.7)	84 (43.8)	0.957
Mental health literacy, M (SD)	3.54 (0.61)	3.57 (0.63)	3.52 (0.60)	3.56 (0.62)	0.882
Psychological distress, M (SD)	2.61 (0.78)	2.58 (0.76)	2.64 (0.79)	2.60 (0.77)	0.904

GenAI, generative artificial intelligence; M, mean; SD, standard deviation. *p* values are from one-way ANOVA or chi-square tests across conditions.

### 3.2 Manipulation checks and group means

The manipulation checks supported the effectiveness of the two safety-design cues. Participants in the privacy-assurance conditions reported significantly higher perceived privacy protection than those in conditions without privacy assurance, *F*(1, 764) = 159.30, *p* < 0.001, ηp^2^ = 0.172, *d* = 0.91. Participants in the professional-boundary warning conditions reported significantly higher boundary awareness than those in conditions without boundary warning, *F*(1, 764) = 176.40, *p* < 0.001, ηp^2^ = 0.188, *d* = 0.96. The interaction between privacy assurance and boundary warning on calibrated trust was also significant, *F*(1, 764) = 9.50, *p* = 0.002, ηp^2^ = 0.012, indicating that calibrated trust was highest when both cues were present. Group-level means and standard deviations are reported in Table 2.

**Table 2:** Group means and standard deviations for focal variables.

Variable	G1 no cues	G2 privacy only	G3 boundary only	G4 both cues
Perceived privacy protection	3.34 ± 0.72	3.92 ± 0.67	3.39 ± 0.71	4.04 ± 0.64
Boundary awareness	3.36 ± 0.70	3.38 ± 0.69	3.97 ± 0.66	4.05 ± 0.63
Calibrated trust	3.34 ± 0.68	3.55 ± 0.65	3.49 ± 0.64	3.98 ± 0.61
Safe-use intention	3.28 ± 0.73	3.66 ± 0.69	3.48 ± 0.70	4.00 ± 0.64
Overreliance risk	3.10 ± 0.74	3.03 ± 0.73	2.66 ± 0.72	2.42 ± 0.70
Professional help-seeking intention	3.34 ± 0.72	3.39 ± 0.70	3.77 ± 0.68	3.96 ± 0.64

Values are means ± standard deviations. G1, no safety cue; G2, privacy assurance only; G3, professional-boundary warning only; G4, both safety cues.

### 3.3 Descriptive statistics, correlations, and measurement model

The correlations among the six focal constructs followed the expected pattern. Perceived privacy protection correlated positively with calibrated trust and safe-use intention. Boundary awareness correlated positively with calibrated trust and professional help-seeking intention, and negatively with overreliance risk. Calibrated trust correlated positively with safe-use intention and negatively with overreliance risk. These correlations supported the theoretical distinction between responsible use and overreliance risk. Full correlations and HTMT information are reported in Supplementary Table S4.

The six-factor CFA model showed good fit to the data, χ^2^(284) = 694.28, CFI = 0.958, TLI = 0.952, RMSEA = 0.043, 90% CI [0.039,0.047], and SRMR = 0.041. Standardized factor loadings ranged from 0.71 to 0.88. Cronbach's alpha values ranged from 0.86 to 0.91, McDonald's omega values ranged from 0.87 to 0.92, CR values ranged from 0.87 to 0.91, and AVE values ranged from 0.62 to 0.71. HTMT values were below 0.85, supporting discriminant validity. Table 3 summarizes the measurement quality indicators.

**Table 3:** Reliability and validity indicators for focal constructs.

Construct	Items	α	ω	CR	AVE	Loading range
Perceived privacy protection	4	0.89	0.90	0.90	0.69	0.78–0.86
Boundary awareness	5	0.88	0.89	0.89	0.62	0.71–0.83
Calibrated trust	5	0.91	0.92	0.91	0.68	0.76–0.87
Safe-use intention	4	0.90	0.91	0.91	0.71	0.80–0.88
Overreliance risk	4	0.86	0.87	0.87	0.63	0.72–0.83
Professional help-seeking intention	4	0.88	0.89	0.89	0.67	0.76–0.86

α, Cronbach's alpha; ω, McDonald's omega; CR, composite reliability; AVE, average variance extracted. HTMT values are reported in Supplementary Table S4.

Common method variance was evaluated using three diagnostic checks. Harman's single-factor test showed that the first unrotated factor explained 27.8% of the variance, below the conventional 50% threshold. A single-factor CFA model showed poor fit, χ^2^(299) = 2790.35, CFI = 0.598, RMSEA = 0.104, and SRMR = 0.096. In addition, full collinearity VIF values ranged from 1.10 to 2.42. These results suggested that common method bias was unlikely to account for the observed associations.

### 3.4 Factorial ANOVA results

The factorial analyses showed distinct effects of the two safety cues. Privacy assurance had a large effect on perceived privacy protection, but not on boundary awareness. Professional-boundary warning had a large effect on boundary awareness, but not on perceived privacy protection. Both cues had positive effects on calibrated trust and safe-use intention. Boundary warning had the strongest effect on overreliance risk and professional help-seeking intention. Table 4 summarizes the factorial ANOVA results.

**Table 4:** Factorial ANOVA results.

Outcome	Effect	*F*	*p*	ηp^2^
Perceived privacy protection	Privacy assurance	159.30	< 0.001	0.172
Perceived privacy protection	Boundary warning	2.10	0.147	0.003
Perceived privacy protection	Privacy × Boundary	0.40	0.527	0.001
Boundary awareness	Privacy assurance	1.80	0.180	0.002
Boundary awareness	Boundary warning	176.40	< 0.001	0.188
Boundary awareness	Privacy × Boundary	0.70	0.403	0.001
Calibrated trust	Privacy assurance	58.20	< 0.001	0.071
Calibrated trust	Boundary warning	42.40	< 0.001	0.053
Calibrated trust	Privacy × Boundary	9.50	0.002	0.012
Safe-use intention	Privacy assurance	84.60	< 0.001	0.100
Safe-use intention	Boundary warning	31.30	< 0.001	0.039
Safe-use intention	Privacy × Boundary	4.20	0.041	0.005
Overreliance risk	Privacy assurance	7.60	0.006	0.010
Overreliance risk	Boundary warning	108.50	< 0.001	0.124
Overreliance risk	Privacy × Boundary	2.10	0.148	0.003
Professional help-seeking intention	Privacy assurance	8.20	0.004	0.011
Professional help-seeking intention	Boundary warning	111.20	< 0.001	0.127
Professional help-seeking intention	Privacy × Boundary	4.60	0.032	0.006

For all factorial effects, df = 1, 764. ηp^2^, partial eta squared. Privacy × Boundary, interaction between privacy assurance and professional-boundary warning.

### 3.5 SEM path analysis and indirect effects

The structural model showed good fit, χ^2^(302) = 742.65, CFI =0.955, TLI = 0.948, RMSEA = 0.044, 90% CI [0.040, 0.048], and SRMR = 0.046. Privacy assurance was associated with higher perceived privacy protection, β = 0.50, *p* < 0.001, and professional-boundary warning was associated with higher boundary awareness, β = 0.53, *p* < 0.001. Perceived privacy protection was associated with calibrated trust, β = 0.29, *p* < 0.001, and safe-use intention, β = 0.25, *p* < 0.001. Boundary awareness was associated with calibrated trust, β = 0.25, *p* < 0.001, lower overreliance risk, β = −0.35, *p* < 0.001, and stronger professional help-seeking intention, β = 0.35, *p* < 0.001. Calibrated trust was associated with safe-use intention, β = 0.42, *p* < 0.001, lower overreliance risk, β = −0.14, *p* = 0.006, and higher professional help-seeking intention, β = 0.18, *p* < 0.001. The privacy assurance × boundary warning interaction was associated with calibrated trust, β = 0.12, *p* = 0.003. Calibrated trust was highest in the both-cues condition (M = 3.98, 95% CI [3.89, 4.07]) compared with privacy assurance only (M = 3.55, 95% CI [3.46, 3.64]), boundary warning only (M = 3.49, 95% CI [3.40, 3.58]), and no cues (M = 3.34, 95% CI [3.24, 3.44]).

**Figure 2:**
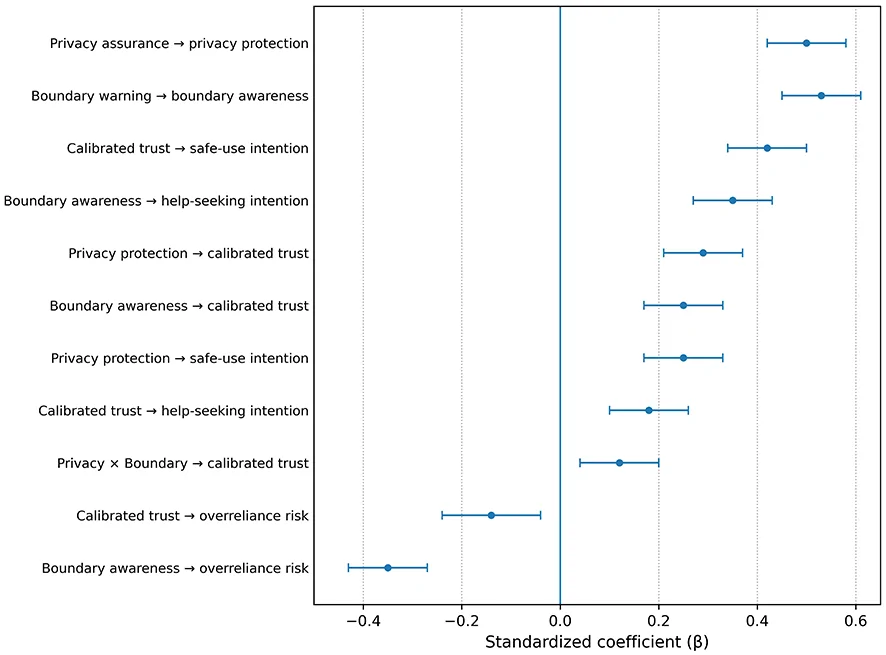
Standardized path coefficients of the structural model. Points indicate standardized coefficients, horizontal bars indicate 95% confidence intervals, and the vertical line represents zero.

**Figure 3:**
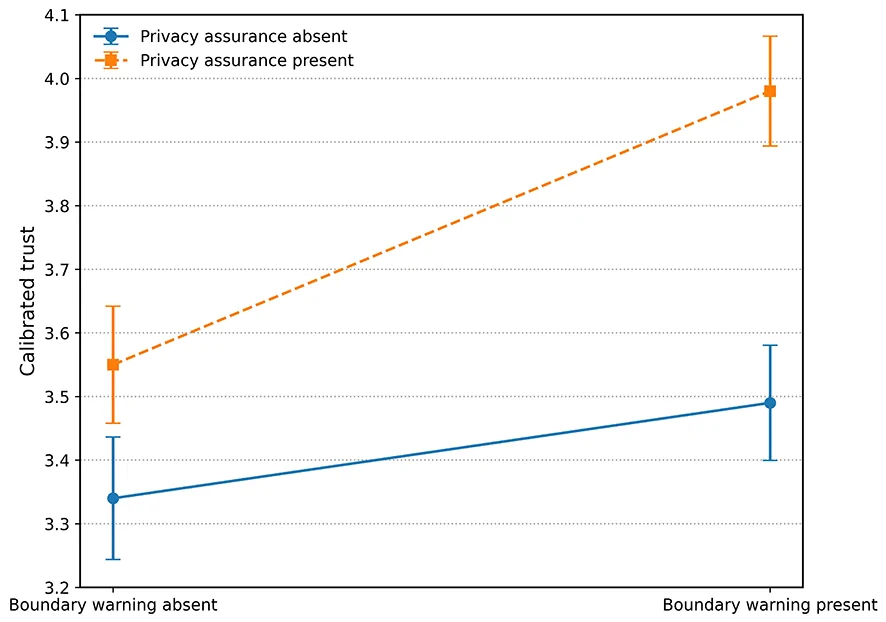
Interaction between privacy assurance and professional-boundary warning on calibrated trust. The figure shows mean levels of calibrated trust across professional-boundary warning conditions separately for participants with and without privacy assurance. Error bars represent 95% confidence intervals.

The model explained 25% of variance in perceived privacy protection, 28% in boundary awareness, 39% in calibrated trust, 50% in safe-use intention, 30% in overreliance risk, and 34% in professional help-seeking intention. The primary indirect effects were significant. Privacy assurance was linked to safe-use intention through perceived privacy protection and calibrated trust, whereas professional-boundary warning was linked to lower overreliance risk and stronger professional help-seeking intention through boundary awareness. Inspection of standardized residuals and modification indices did not indicate localized misfit requiring post hoc structural paths. The standardized path coefficients are illustrated in Figure 2, and detailed estimates are reported in Table 5. The interaction pattern for calibrated trust is presented in Figure 3.

**Table 5:** SEM path coefficients and key indirect effects.

Path/indirect effect	Estimate	SE	95% CI	*p*
Privacy assurance → perceived privacy protection	0.50	0.04	[0.42, 0.58]	< 0.001
Boundary warning → boundary awareness	0.53	0.04	[0.45, 0.61]	< 0.001
Perceived privacy protection → calibrated trust	0.29	0.04	[0.21, 0.37]	< 0.001
Boundary awareness → calibrated trust	0.25	0.04	[0.17, 0.33]	< 0.001
Privacy × Boundary → calibrated trust	0.12	0.04	[0.04, 0.20]	0.003
Calibrated trust → safe-use intention	0.42	0.04	[0.34, 0.50]	< 0.001
Privacy protection → safe-use intention	0.25	0.04	[0.17, 0.33]	< 0.001
Boundary awareness → overreliance risk	−0.35	0.04	[−0.43, −0.27]	< 0.001
Calibrated trust → overreliance risk	−0.14	0.05	[−0.24, −0.04]	0.006
Boundary awareness → help-seeking intention	0.35	0.04	[0.27, 0.43]	< 0.001
Calibrated trust → help-seeking intention	0.18	0.04	[0.10, 0.26]	< 0.001
Privacy → privacy protection → safe-use intention	0.125	0.026	[0.078, 0.181]	–
Privacy → privacy protection → calibrated trust → safe-use intention	0.061	0.018	[0.031, 0.103]	–
Boundary → boundary awareness → overreliance risk	−0.186	0.033	[−0.254, −0.128]	–
Boundary → boundary awareness → help-seeking intention	0.186	0.032	[0.126, 0.253]	–

β, standardized coefficient; SE, standard error; CI, confidence interval. Direct effects are standardized estimates. Indirect effects were estimated using bootstrap resampling; *p* values are not reported for indirect effects because significance was determined by 95% bootstrap confidence intervals excluding zero.

### 3.6 Robustness checks

The findings were stable across robustness checks. Re-estimating the structural model without covariates did not change the direction or significance of the core paths, and standardized coefficients changed by less than 0.03. Robust maximum likelihood estimation produced similar model fit, CFI = 0.953, RMSEA = 0.045, SRMR = 0.047. WLSMV estimation for ordinal indicators yielded the same pattern of significant main pathways. Excluding frequent GenAI users did not alter the main effects of privacy assurance and boundary warning. Restricting the analysis to participants who passed the manipulation checks also preserved the direction and significance of the core pathways. Bootstrapping with 10,000 resamples confirmed that the primary indirect effects had confidence intervals excluding zero. Gender measurement invariance was examined for the six-factor measurement model. Configural, metric, and scalar models showed acceptable fit, and changes in CFI were below 0.010 and changes in RMSEA were below 0.015, supporting measurement invariance across gender groups. Robustness checks and measurement invariance results are reported in Supplementary Tables S5, S6.

## 4 Discussion

### 4.1 Principal findings

Because this study used hypothetical vignette scenarios rather than actual chatbot interactions, the findings should be interpreted as evidence about students' safety-related evaluations and intentions, not as evidence of real-world chatbot use or clinical help-seeking behavior. Within that boundary, the study examined whether two interface messages, privacy assurance and professional-boundary warning, shape students' evaluations of generative AI mental health chatbots. The results supported the proposed model. Students noticed the difference between the two messages: one addressed the safety of disclosure, whereas the other clarified the limits of reliance.

Trust calibration appeared to be an important bridge between the messages and appropriate use. Perceived privacy protection and boundary awareness were both associated with calibrated trust, which in turn was linked to safe-use intention. The point is not to make students trust AI chatbots more in general; rather, the results suggest that trust is more useful when it is accompanied by clear information about what the system can and cannot do.

The boundary message was especially relevant to risk-sensitive outcomes. Greater boundary awareness was linked to lower overreliance risk and stronger intention to seek professional help. This pattern suggests that professional-limit statements may do more than protect organizations from liability. When framed clearly, they may help users view a chatbot as a possible first step, not as a replacement for qualified care.

The interaction result further suggests that the two messages work best together. A privacy assurance alone may make the tool easier to approach, but it does not by itself clarify when human care is needed. A boundary warning alone may limit inappropriate reliance, but it does not fully address disclosure concerns. When both messages are present, users receive a more complete account of the chatbot's role: information may be protected, but the tool remains limited and professionally bounded.

### 4.2 Theoretical implications

The study contributes to psychological research on AI in three ways. First, it shifts the focus from technology acceptance to safety design. Much existing research on AI chatbot use examines whether students accept, adopt, or intend to continue using such tools. That work is valuable, but mental health-related contexts require additional attention to privacy, professional boundaries, and overreliance. The present study shows that responsible use can be conceptualized as a multidimensional outcome involving safe-use intention, reduced overreliance, and professional help-seeking intention.

Second, the study clarifies the role of trust. In many technology acceptance models, trust is treated as a straightforward positive predictor of behavioral intention. In mental health-related AI support, however, more trust is not necessarily better. Excessive or poorly calibrated trust may lead users to follow AI-generated suggestions beyond their appropriate scope. By using calibrated trust as the focal mechanism, the study links human-AI trust research with health psychology concerns about safety, disclosure, and appropriate care pathways.

Third, the study offers an experimental account of how interface-level cues shape psychological responses. Rather than measuring privacy concerns and trust as stable attitudes, the study manipulated concrete safety cues that could be implemented in chatbot introductions, onboarding screens, or institutional guidelines. This supports a design-oriented psychological approach to trustworthy AI: users' trust and reliance are not only individual dispositions but also responses to the information environment created by system designers and institutions.

### 4.3 Practical implications

The findings have implications for universities, developers, and student support services. First, privacy notices should be concrete and placed before students disclose sensitive information. Rather than using broad assurances, interfaces should state what information is collected, what is not collected, whether conversation content is stored, who can access it, how long it is retained, and how users can stop or delete a session where applicable. Such information should be written in plain language and displayed before the chatbot invites mental health-related disclosure.

Second, professional-boundary warnings should be treated as a core safety feature rather than as a legal disclaimer hidden in terms of service. A responsible chatbot should clearly state at onboarding that it cannot diagnose mental disorders, provide psychotherapy, replace counselors or physicians, or handle emergencies. Boundary messages should also appear again when users mention severe distress, self-harm, harm to others, medical decisions, or prolonged functional impairment. In these moments, the system should provide referral pathways to campus counseling, qualified professionals, medical institutions, or emergency services.

Third, universities should develop institutional policies that define AI chatbots as educational and support-navigation tools, not as counseling services. Such policies should specify approved use cases, referral procedures, emergency escalation routes, privacy responsibilities, staff oversight, and communication materials for students. Digital health literacy training should therefore emphasize not only how to use generative AI effectively, but also when not to rely on it as the primary source of support.

### 4.4 Limitations

Several limitations should be acknowledged. First, the study used a vignette experiment rather than observation of actual chatbot interactions. Vignettes allow strong control over design cues, but participants' responses may differ from behavior during real-time conversation with an AI system. Future work should test similar safety cues in functioning chatbot interfaces and examine behavioral outcomes such as click-through to help resources or actual referral use.

Second, the outcomes were self-reported intentions and risk perceptions. Although intentions are meaningful in health psychology, they do not perfectly predict behavior. Future studies could combine self-report with behavioral tasks, interaction logs, or longitudinal follow-up. For example, researchers could examine whether boundary warnings affect whether users choose professional referral information after receiving a simulated chatbot response.

Third, the sample consisted of college students in one national context. The findings should be interpreted within the cultural and institutional context in which the study was conducted. Students' responses to privacy messages, professional-boundary warnings, and help-seeking recommendations may vary across countries because of differences in mental health stigma, campus counseling systems, data-protection norms, trust in institutions, and familiarity with AI tools. Cross-cultural studies and multi-institutional samples would help determine whether the safety-design mechanisms generalize across different higher education systems.

Fourth, the study examined two specific safety cues. Other design features may also shape responsible use, including uncertainty labels, human escalation buttons, referral prompts, crisis detection messages, source citations, and model limitations disclosures. Future research should compare these features and identify combinations that promote calibrated trust without discouraging appropriate help-seeking.

### 4.5 Future research

Future studies should extend the present design in several directions. Longitudinal research could examine whether repeated exposure to privacy and boundary cues produces stable changes in trust calibration. Field experiments could evaluate whether safety cues embedded in real university chatbot services increase responsible use and referral behavior. Experimental studies could also compare different message tones, such as supportive, neutral, or risk-emphasizing boundary warnings, to determine how warnings can reduce overreliance without creating unnecessary fear.

Another important direction is the study of high-risk contexts. The present study did not provide clinical support and did not test crisis intervention. Future research should work closely with clinicians, ethicists, and campus mental health services to evaluate how AI systems can safely redirect users to qualified help when severe distress, self-harm risk, or emergency needs are detected. Recent safety-monitoring and risk-mitigation work suggests that technical safeguards alone are insufficient without structured crisis protocols, human oversight, and clear institutional responsibilities (Olisaeloka et al., 2026; Pichowicz et al., 2025; Szoke et al., 2026). Such research should prioritize safety, human oversight, and professional accountability.

## 5 Conclusion

This study shows that two simple interface messages–privacy assurance and professional-boundary warning–can shape how students evaluate generative AI mental health chatbots. Privacy assurance made sensitive disclosure feel safer, while the boundary message clarified that chatbot output cannot replace qualified care. The principal practical contribution is that these messages work best when used together: privacy information supports safer disclosure, and boundary information supports more appropriate reliance. Together, the two messages were linked to more appropriately calibrated trust, lower overreliance risk, and stronger professional help-seeking intention. For AI tools in mental health-related contexts, the design goal should not be to encourage use without qualification. It should be to make the conditions, limits, and referral pathways of use clear enough for students to treat chatbots as educational and support-navigation resources rather than substitutes for professional services.

## Data Availability

The raw data supporting the conclusions of this article will be made available by the authors, without undue reservation.
