# Supplementary material for: Privacy assurances and professional-boundary warnings in generative AI mental health chatbots: a randomized vignette experiment on calibrated trust, overreliance risk, and professional help-seeking intentions
Source: Front Psychol. 2026 Sep 1;17:1934264. doi: 10.3389/fpsyg.2026.1934264 (PMC13575988; doi:10.3389/fpsyg.2026.1934264)
Supplement: Supplementary file 1 [file Table_1.docx]

# Supplementary Material

Table S1. Full questionnaire, manipulation-check, and covariate items.

| **Construct** | **Item** | **Wording** |
| --- | --- | --- |
| Perceived privacy protection | PPP1 | The chatbot clearly explained how users' mental health-related information would be protected. |
| Perceived privacy protection | PPP2 | The chatbot appeared to provide sufficient privacy protection for users. |
| Perceived privacy protection | PPP3 | I understood how my information would be handled when using the chatbot. |
| Perceived privacy protection | PPP4 | I would feel that my sensitive information was handled carefully in this chatbot. |
| Boundary awareness | BA1 | The chatbot clearly stated that it was not a substitute for professional mental health care. |
| Boundary awareness | BA2 | The chatbot made clear when users should seek help from qualified professionals. |
| Boundary awareness | BA3 | The chatbot clearly explained its limits in mental health support. |
| Boundary awareness | BA4 | I understood that the chatbot could not provide diagnosis, psychotherapy, or crisis intervention. |
| Boundary awareness | BA5 | I understood that serious distress requires professional support rather than relying only on AI. |
| Calibrated trust | CT1 | I would trust the chatbot for general mental health information within its limits. |
| Calibrated trust | CT2 | I would use the chatbot while keeping in mind that it cannot replace a professional. |
| Calibrated trust | CT3 | The chatbot seems useful for low-threshold support but limited for serious problems. |
| Calibrated trust | CT4 | I could benefit from the chatbot if I used its advice carefully. |
| Calibrated trust | CT5 | My trust in the chatbot would depend on whether its limits were clear. |
| Safe-use intention | SUI1 | I would use the chatbot as a supplementary source of general support. |
| Safe-use intention | SUI2 | I would use the chatbot for initial coping suggestions when appropriate. |
| Safe-use intention | SUI3 | I would use the chatbot to learn about possible help-seeking resources. |
| Safe-use intention | SUI4 | I would use the chatbot only within clearly appropriate limits. |
| Overreliance risk | ORR1 | I might rely on the chatbot even when professional help would be more appropriate. |
| Overreliance risk | ORR2 | I might follow the chatbot's suggestions without checking with a qualified professional. |
| Overreliance risk | ORR3 | I might delay seeking professional help if the chatbot seemed helpful. |
| Overreliance risk | ORR4 | I might treat the chatbot's responses as professional mental health advice. |
| Professional help-seeking intention | PHS1 | I would seek help from a qualified professional if my distress became severe. |
| Professional help-seeking intention | PHS2 | I would contact campus counseling services when AI support is insufficient. |
| Professional help-seeking intention | PHS3 | I would use the chatbot as a first step rather than a replacement for professional help. |
| Professional help-seeking intention | PHS4 | I would follow referral information provided by the chatbot when professional help is needed. |
| Privacy manipulation check | PMC1 | The vignette clearly explained how users' mental health-related information would be protected. |
| Privacy manipulation check | PMC2 | The vignette clearly explained how user information would be handled. |
| Privacy manipulation check | PMC3 | The chatbot appeared to provide sufficient privacy safeguards. |
| Boundary manipulation check | BMC1 | The vignette clearly stated that the chatbot was not a substitute for professional mental health care. |
| Boundary manipulation check | BMC2 | The vignette made clear when users should seek help from qualified professionals. |
| Boundary manipulation check | BMC3 | The chatbot clearly explained its limits in mental health support. |
| Prior GenAI use | PGU1 | How often have you used generative AI tools before this study? |
| Prior mental health education | PMHE1 | Have you previously received formal mental health education or training? |
| Mental health literacy (covariate) | MHL1 | I know how to recognize signs of common mental health problems. |
| Mental health literacy (covariate) | MHL2 | I know where students can seek professional help for mental health problems. |
| Mental health literacy (covariate) | MHL3 | I know how to evaluate whether mental health information is reliable. |
| Mental health literacy (covariate) | MHL4 | I know when emotional distress requires professional support. |
| Psychological distress (covariate) | PD1 | During the past two weeks, I felt nervous or anxious. |
| Psychological distress (covariate) | PD2 | During the past two weeks, I felt down or hopeless. |
| Psychological distress (covariate) | PD3 | During the past two weeks, I had difficulty sleeping because of distress. |
| Psychological distress (covariate) | PD4 | During the past two weeks, I found it hard to concentrate because of distress. |

Note. Focal construct, manipulation-check, mental health literacy, and psychological distress items used 5-point Likert-type response scales unless otherwise stated. Demographic covariates were assessed with categorical or numeric response formats as appropriate.

**Table S2. Vignette texts**

| **Component** | **Text** |
| --- | --- |
| Base vignette | Imagine that your university provides access to a generative AI chatbot. The chatbot can answer general questions about stress, sleep, study pressure, emotional regulation, and help-seeking resources. It can provide general information, coping suggestions, emotional reassurance, and guidance on how to seek support. It does not provide clinical diagnosis, psychotherapy, crisis intervention, or medical treatment. |
| Privacy assurance add-on | Before use, the chatbot clearly explains that it minimizes the collection of personal information, protects mental health-related data, avoids using identifiable information for unrelated purposes, and explains how user information is handled. |
| Professional-boundary warning add-on | Before use, the chatbot clearly explains that it is not a substitute for professional mental health care, clinical diagnosis, psychotherapy, crisis intervention, or emergency services. It advises users to contact qualified counselors, medical professionals, campus counseling services, or emergency services when distress is severe or urgent. |
| G1: no privacy assurance + no boundary warning | Participants saw only the base vignette. |
| G2: privacy assurance only | Participants saw the base vignette plus the privacy assurance add-on. |
| G3: boundary warning only | Participants saw the base vignette plus the professional-boundary warning add-on. |
| G4: both cues | Participants saw the base vignette plus both add-ons. |

**Table S3. Exclusion criteria and data-cleaning flow**

| **Exclusion reason** | **n** |
| --- | --- |
| No informed consent | 7 |
| Age below 18 years | 9 |
| Not currently enrolled in college | 8 |
| Failed attention check | 19 |
| Completion time below 180 seconds | 13 |
| Straight-lining or patterned responses | 8 |
| Total excluded | 64 |
| Final analytic sample | 768 |

**Table S4. Correlations and discriminant validity**

| **Variable** | **1** | **2** | **3** | **4** | **5** | **6** |
| --- | --- | --- | --- | --- | --- | --- |
| 1. Perceived privacy protection | -- |  |  |  |  |  |
| 2. Boundary awareness | .18*** | -- |  |  |  |  |
| 3. Calibrated trust | .42*** | .38*** | -- |  |  |  |
| 4. Safe-use intention | .46*** | .31*** | .56*** | -- |  |  |
| 5. Overreliance risk | -.23*** | -.41*** | -.19*** | -.13** | -- |  |
| 6. Professional help-seeking intention | .26*** | .44*** | .27*** | .36*** | -.23*** | -- |

*Note. Values below the diagonal are Pearson correlations. HTMT values were below .85 for all construct pairs. ** p < .01; *** p < .001.*

**Table S5. Robustness checks**

| Robustness check | Result |
| --- | --- |
| Model without covariates | Core paths retained the same direction and significance; standardized coefficients changed by less than .03. |
| Covariate-adjusted ANCOVA | Main and interaction effects showed the same pattern as the primary factorial ANOVA results. |
| Robust maximum likelihood estimation | Model fit remained acceptable: CFI = .953, RMSEA = .045, SRMR = .047. |
| WLSMV estimation for ordinal indicators | Main paths remained significant and directionally consistent. |
| Excluding frequent generative AI users | Privacy and boundary-warning effects remained significant; standardized coefficients changed by less than .04. |
| Participants passing manipulation checks only | The direction and significance of the core pathways remained unchanged. |
| Bootstrap resampling | Indirect-effect confidence intervals based on 10,000 resamples did not include zero. |
| Gender measurement invariance and multi-group sensitivity analysis | Configural, metric, and scalar invariance models supported measurement invariance across gender groups; key structural paths did not show meaningful group differences. |

Table S6. Gender measurement invariance results

| Model | χ² | df | CFI | TLI | RMSEA | SRMR | ΔCFI | ΔRMSEA |
| --- | --- | --- | --- | --- | --- | --- | --- | --- |
| Configural invariance | 1218.64 | 568 | .956 | .950 | .039 | .045 | -- | -- |
| Metric invariance | 1245.37 | 588 | .956 | .951 | .038 | .048 | .000 | -.001 |
| Scalar invariance | 1281.92 | 608 | .955 | .952 | .038 | .050 | -.001 | .000 |

Note. Gender groups were coded according to participants' self-reported gender. ΔCFI and ΔRMSEA were evaluated relative to the less constrained model. Values of ΔCFI < .010 and ΔRMSEA < .015 were interpreted as supporting invariance.

**Figure S1. Participant screening and experimental allocation flow.**

*Note. The figure shows the flow of responses from initial collection to the final analytic sample and the random allocation of eligible participants to the four vignette conditions. G1 = no cues; G2 = privacy assurance only; G3 = professional-boundary warning only; G4 = both cues.*
